# Sociodemographic Variation in Early Uptake of COVID-19 Vaccine and Parental Intent and Attitudes Toward Vaccination of Children Aged 6 Months–4 Years — United States, July 1–29, 2022

**DOI:** 10.15585/mmwr.mm7146a3

**Published:** 2022-11-18

**Authors:** Tammy A. Santibanez, Tianyi Zhou, Carla L. Black, Tara M. Vogt, Bhavini Patel Murthy, Vicki Pineau, James A. Singleton

**Affiliations:** ^1^Immunization Services, National Center for Immunization and Respiratory Diseases, CDC; ^2^Leidos Health Inc., Atlanta, Georgia; ^3^NORC at the University of Chicago, Chicago, Illinois.

COVID-19 vaccines are safe and effective for infants and young children, and on June 18, 2022, CDC recommended COVID-19 vaccination for infants and children (children) aged 6 months–4 years (*1*,*2*). As of November 9, 2022, based on administrative data reported to CDC,* 5.9% of children aged <2 years and 8.8% of children aged 2–4 years had received ≥1 dose. To better understand reasons for low coverage among children aged <5 years, CDC analyzed data from 4,496 National Immunization Survey-Child COVID Module (NIS-CCM) interviews conducted during July 1–29, 2022, to examine variation in receipt of ≥1 dose of COVID-19 vaccine and parental intent to vaccinate children aged 6 months–4 years by sociodemographic characteristics and by parental beliefs about COVID-19; type of vaccination place was also reported. Among children aged 6 months–4 years, 3.5% were vaccinated; 59.3% were unvaccinated, but the parent was open to vaccination; and 37.2% were unvaccinated, and the parent was reluctant to vaccinate their child. Openness to vaccination was higher among parents of Hispanic or Latino (Hispanic) (66.2%), non-Hispanic Black or African American (Black) (61.1%), and non-Hispanic Asian (Asian) (83.1%) children than among parents of non-Hispanic White (White) (52.9%) children and lower among parents of children in rural areas (45.8%) than among parents of children in urban areas (64.1%). Parental confidence in COVID-19 vaccine safety and receipt of a provider recommendation for COVID-19 vaccination were lower among unvaccinated than vaccinated children. COVID-19 vaccine recommendations from a health care provider, along with dissemination of information about the safety of COVID-19 vaccine by trusted persons, could increase vaccination coverage among young children.

NIS-CCM^†^ is an ongoing, national random-digit–dialed mobile telephone survey of households that include children and adolescents aged 6 months–17 years (*3*,*4*). The survey respondent was a parent or guardian (parent) who indicated they were knowledgeable about the child’s vaccination history. COVID-19 vaccination status was based on the parent’s response to the question, “Has [child] received at least one dose of a COVID-19 vaccine?” Among parents of unvaccinated children, parental intent was measured by asking, “How likely are you to get [child] a COVID-19 vaccine? Would you say you would definitely get a vaccine for [child], probably get a vaccine, probably not get a vaccine, definitely not get a vaccine, or are not sure?” Responses were grouped as follows: 1) child vaccinated with ≥1 dose; 2) child unvaccinated and parent open to vaccination, defined as parents of unvaccinated children reporting they definitely or probably would get the child vaccinated or were unsure; and 3) child unvaccinated and parent reluctant to vaccinate, defined as parents of unvaccinated children reporting they definitely or probably would not get the child vaccinated. Parents of vaccinated children also reported the type of place^§^ where the child was vaccinated. Variables^¶^ describing potential drivers of COVID-19 vaccine acceptance were derived from the Behavioral and Social Drivers of Vaccination (BeSD) framework (*5*).

Data from 4,496 interviews conducted during July 1–29, 2022, were analyzed; estimating parental intent to vaccinate their child began during December 2021.** The cumulative NIS-CCM response rate was 20.4%. Weighted proportions with 95% CIs were estimated, accounting for the complex survey design and weights, using SUDAAN (version 11.0.3; RTI International) and SAS (version 9.4; SAS Institute, Inc.).^††^ T-tests for proportions were used to test for differences, with p-values <0.05 considered statistically significant. This activity was reviewed by CDC and was conducted consistent with applicable federal law and CDC policy.^§§^

## National COVID-19 Vaccination Coverage, Parental Intent, and Place of Vaccination

**FIGURE 1:**
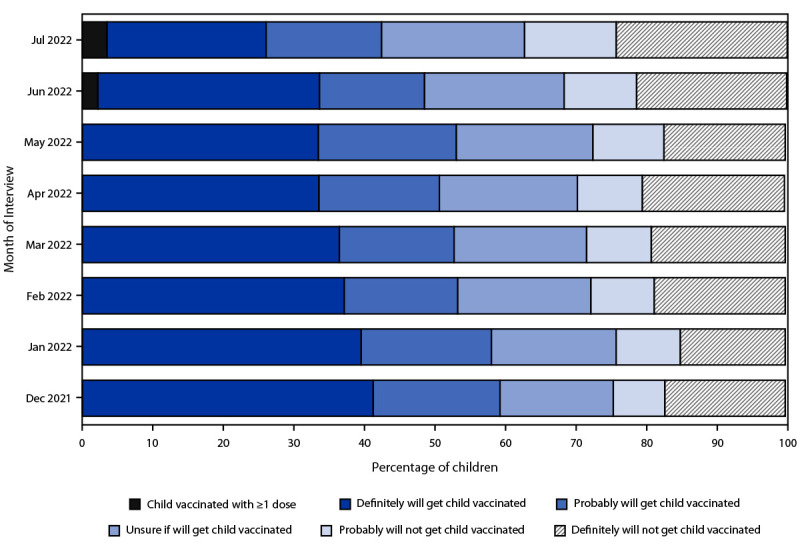
Children’s COVID-19 vaccination status and parental intent to have unvaccinated children aged 6 months–4 years vaccinated — National Immunization Survey-Child COVID Module, United States, December 2021–July 2022*^,^^†^ * The June 2022 estimate of the percentage of children aged 6 months–4 years who had received ≥1 COVID-19 vaccine dose was not calibrated to administrative data; the estimate is an overestimate of coverage and should be interpreted with caution. The estimate was retained so that the percentages would sum to 100%. ^†^ The recommendation for COVID-19 vaccination for this age group began on June 18, 2022. Estimates for July 2022 are from interviews conducted during July 1–29, 2022. Estimates for December 2021–June 2022 are from National Immunization Survey-Child COVID Module interviews conducted during November 28, 2021–June 30, 2022.

During the period preceding authorization of the vaccine for children aged 6 months–4 years, the percentage of children whose parent reported they definitely would get their child vaccinated decreased from 41.3% in December 2021 to 33.5% in May 2022 (Figure 1). By mid-July 2022, 3.5% of children were reported to have received ≥1 dose, 59.3% were unvaccinated and the parent reported being open to vaccination (22.6% definitely would, 16.4% probably would, and 20.3% were unsure), and 37.2% were unvaccinated and the parent was reluctant to vaccinate (13.0% probably would not and 24.3% definitely would not) (Figure 1). The distribution of places where vaccination was received was 78.5% in a medical setting (40.0% doctor’s office, 21.1% clinic or health center, 11.4% hospital, 5.0% health department, and 1.0% other medical place); 15.0% at a pharmacy or drug store; 4.4% at a mass vaccination place; 1.8% at another nonmedical place; and 0.3% at a school.

## Vaccination Coverage and Parental Intent, by Selected Characteristics

A higher percentage of White children (4.5%) had received COVID-19 vaccination during the first month of recommendation than Hispanic (2.5%) and Black children (1.0%) (Table). However, higher percentages of Asian (83.1%), Black (61.1%), and Hispanic (66.2%) children had parents reporting they were open to vaccination compared with White children (52.9%). A higher percentage of children in households with income >$75,000 per year and with higher maternal educational attainment had received ≥1 dose of COVID-19 vaccine versus children from lower-income households and those whose mothers had lower educational attainment. A lower percentage of children living in rural areas had been vaccinated (1.6%) compared with those living in urban areas (4.2%), and a lower percentage of children living in rural areas (45.8%) had parents reporting they were open to getting their child vaccinated compared with those living in urban areas (64.1%). The percentage of children who received COVID-19 vaccination and the percentage of children with parents reporting they were reluctant to get their child vaccinated varied by U.S. Department of Health and Human Services (HHS) region (range = 1.6%–7.3% and 26.2%–48.4%), respectively.

**TABLE:** COVID-19 vaccination status and intention of parents to vaccinate children aged 6 months–4 years, by sociodemographic characteristics — National Immunization Survey-Child COVID Module, United States, July 1–29, 2022

Characteristic	Total no.	Children, % (95% CI)*		
		Vaccinated with ≥1 dose	Unvaccinated, parent open to vaccination^†^	Unvaccinated, parent reluctant to vaccinate^§^
**Overall**	**4,496**	**3.5 (3.0–4.1)**	**59.3 (56.7–61.8)**	**37.2 (34.8–39.8)**
**Age group**				
6 mos–1 yr (Ref)	**1,383**	3.0 (2.2–4.0)	58.7 (54.1–63.2)	38.3 (33.8–43.0)
2–4 yrs	**3,113**	3.7 (3.1–4.5)	59.5 (56.5–62.5)	36.8 (33.8–39.8)
**Race or ethnicity^¶^**				
Asian	**275**	3.7 (1.8–6.7)	83.1 (76.0–88.9)**	13.1 (7.8–20.3)**
Black or African American	**413**	1.0 (0.3–2.6)**	61.1 (53.8–68.1)**	37.8 (30.9–45.2)
Hispanic or Latino	**956**	2.5 (1.6–3.7)**	66.2 (60.6–71.4)**	31.3 (26.1–36.8)**
White (Ref)	**2,392**	4.5 (3.7–5.4)	52.9 (49.5–56.4)	42.5 (39.1–46.0)
Multiple races or other	**460**	—^††^	52.8 (44.2–61.2)	42.5 (34.0–51.2)
**Sex**				
Male (Ref)	**2,307**	3.4 (2.7–4.3)	60.7 (57.1–64.1)	35.9 (32.4–39.4)
Female	**2,179**	3.5 (2.8–4.4)	57.8 (54.1–61.4)	38.7 (35.1–42.4)
**Household income and poverty level^§§^**				
>$75,000/yr and above poverty level (Ref)	**2,158**	5.3 (4.4–6.3)	60.9 (57.1–64.6)	33.8 (30.1–37.6)
≤$75,000/yr and above poverty level	**1,040**	2.3 (1.5–3.4)**	57.7 (52.5–62.7)	40.0 (35.0–45.1)**
Below poverty level	**401**	1.6 (0.6–3.4)**	54.0 (45.6–62.2)	44.4 (36.2–52.8)**
Not reported	**897**	3.0 (1.9–4.5)**	61.6 (56.2–66.8)	35.4 (30.3–40.8)
**Mother’s education level**				
College degree or higher (Ref)	**2,473**	6.2 (5.3–7.3)	64.8 (61.3–68.2)	29.0 (25.7–32.4)
Some college or vocational school	**1,059**	2.0 (1.2–3.0)**	55.3 (50.6–59.9)**	42.7 (38.1–47.4)**
High school or equivalent	**752**	1.5 (0.7–2.6)**	54.6 (48.9–60.3)**	43.9 (38.3–49.6)**
Less than high school	**212**	2.2 (0.7–5.3)**	60.1 (49.2–70.2)	37.8 (27.7–48.6)
**No. of children and adolescents aged <18 yrs in house**				
1 (Ref)	**2,148**	3.9 (3.1–4.9)	61.7 (57.8–65.5)	34.4 (30.6–38.3)
2–3	**2,117**	3.4 (2.6–4.2)	58.9 (55.4–62.4)	37.7 (34.3–41.2)
≥4	**231**	2.4 (0.9–5.3)	49.1 (38.4–59.9)**	48.4 (37.8–59.2)**
**Ever had COVID-19^¶¶^**				
Yes (Ref)	**1,718**	3.5 (2.6–4.4)	63.9 (60.0–67.7)	32.6 (28.9–36.5)
No	**2,690**	3.6 (2.9–4.4)	56.9 (53.5–60.2)**	39.6 (36.3–42.9)**
Urban-rural residence***				
Urban (MSA, principal city) (Ref)	**1,591**	4.2 (3.3–5.3)	64.1 (59.7–68.3)	31.7 (27.6–36.1)
Suburban (MSA, nonprincipal city)	**2,058**	3.6 (2.8–4.5)	59.4 (55.7–62.9)	37.1 (33.6–40.7)
Rural (non-MSA)	**739**	1.6 (0.8–2.7)**	45.8 (39.4–52.3)**	2.6 (46.1–59.1)**
**SVI of county of residence^†††^**				
Low (Ref)	**1,421**	4.8 (3.8–6.1)	58.0 (53.6–62.3)	37.2 (32.9–41.6)
Moderate	**1,440**	3.5 (2.6–4.5)**	57.6 (53.0–62.1)	39.0 (34.5–43.6)
High	**1,265**	2.9 (2.1–4.0)**	62.8 (58.0–67.3)	34.3 (29.8–39.0)
**HHS region^¶¶¶^**				
1 (Ref)	**390**	7.3 (4.9–10.4)	63.2 (55.2–70.7)	29.5 (22.3–37.6)
2	**316**	3.4 (1.7–6.0)**	61.0 (51.4–70.0)	35.6 (26.7–45.4)
3	**657**	5.5 (3.8–7.7)**	60.8 (54.4–67.0)	33.7 (27.6–40.1)
4	**699**	1.6 (0.8–2.8)**	57.3 (51.6–62.8)	41.1 (35.6–46.8)**
5	**496**	3.3 (1.9–5.2)**	56.4 (49.7–62.9)	40.3 (33.8–47.1)**
6	**722**	2.4 (1.4–3.8)**	56.7 (50.6–62.7)	40.9 (34.9–47.1)**
7	**251**	3.0 (1.3–6.0)**	48.6 (38.9–58.3)**	48.4 (38.8–58.2)**
8	**421**	3.6 (2.1–5.9)**	59.5 (51.7–67.0)	36.9 (29.5–44.7)
9	**338**	4.9 (2.8–7.7)	68.9 (59.7–77.2)	26.2 (18.1–35.6)
10	**206**	5.1 (2.5–9.0)	53.0 (42.4–63.4)	41.9 (31.7–52.7)

**Abbreviations:** HHS = U.S. Department of Health and Human Services; MSA = metropolitan statistical area; Ref = referent group; SVI = social vulnerability index.

* Percentages are for rows.

^†^ Definitely or probably will get child vaccinated or are unsure.

^§^ Definitely or probably will not get child vaccinated.

^¶^ Race of child was reported by parent or guardian respondent. Children who were Asian, Black or African American, multiracial, White, or other races were not Hispanic or Latino (non-Hispanic); children of Hispanic ethnicity might be of any race. Children identified as multiple races had more than one race category selected; “other” race included American Indian or Alaska Native and Native Hawaiian or other Pacific Islander. White race was used as the referent group because of the large sample size, higher vaccination coverage, and lower intent to vaccinate.

** Statistically significant at p<0.05 compared with referent group.

^††^ Estimates not meeting reliability criteria for proportions were suppressed. https://www.cdc.gov/nchs/data/series/sr_02/sr02_175.pdf

^§§^ Household income and poverty level was defined based on total family income for the past calendar year and the U.S. Census Bureau poverty thresholds for that year. https://www.census.gov/data/tables/time-series/demo/income-poverty/historical-poverty-thresholds.html

^¶¶^ On the basis of parent report and not confirmed by COVID-19 testing (respondents not reporting were excluded from the denominator).

*** On the basis of respondent-reported zip code of residence.

^†††^ Categorization of National Immunization Survey-Child COVID Module data into an SVI level was based on respondent-reported zip code of residence. https://www.atsdr.cdc.gov/placeandhealth/svi/index.html

^§§§^ https://www.hhs.gov/about/agencies/iea/regional-offices/index.html

## Attitudes and Social Factors

**FIGURE 2:**
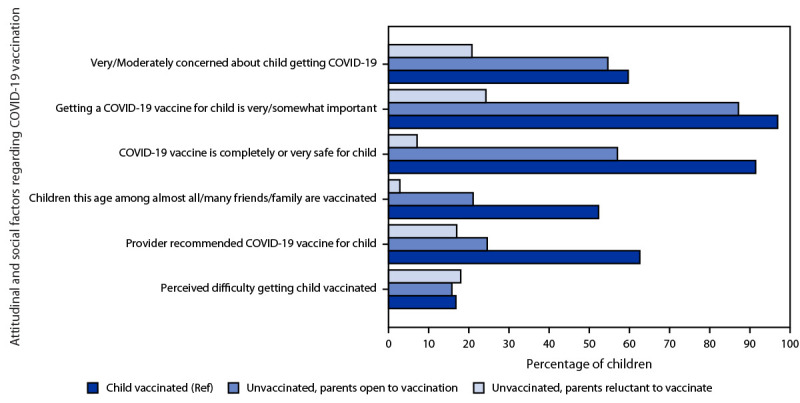
Attitudinal and social factors regarding COVID-19 vaccination, by vaccination status (≥1 dose) and intention of parents to vaccinate children aged 6 months–4 years* — National Immunization Survey-Child COVID Module, United States, July 1–29, 2022 **Abbreviation:** Ref = referent group. * The difference between parents who were reluctant to have their child vaccinated and parents of vaccinated children (Ref) was statistically significant for all factors except perceived difficulty in getting the child vaccinated. The difference between parents who were open to having their child vaccinated and parents of vaccinated children was statistically significant for all factors except 1) concern about the child getting COVID-19, and 2) perceived difficulty in getting the child vaccinated.

A high percentage (87.3%) of unvaccinated children whose parent was open to vaccination had a parent reporting that getting a COVID-19 vaccine for their child was somewhat or very important; however, a lower percentage had parents perceive the vaccine as safe than among vaccinated children (57.1% versus 91.6%), and a lower percentage reported having received a provider recommendation for vaccination (24.6% versus 62.7%) (Figure 2). Among unvaccinated children whose parent was open to vaccination, a lower percentage of Hispanic (47.9%) and Black (47.3%) children’s parents perceived the vaccine as safe compared with White children (66.6%), and a lower percentage of children in households below the poverty level (37.5%) had parents perceiving the vaccine as safe compared with children in households with income >$75,000 per year (69.6%) (Supplementary Table, https://stacks.cdc.gov/view/cdc/122000). Parents reluctant to vaccinate their child were less likely to report being concerned about the child getting COVID-19 than parents of vaccinated children (20.8% versus 59.8%), consider vaccination to be important (24.3% versus 97.1%), consider COVID-19 vaccine to be safe (7.1% versus 91.6%), and report a provider recommendation for COVID-19 vaccination (17.0% versus 62.7%) (Figure 2).

## Discussion

This analysis indicates that 3.5% of children aged 6 months–4 years had received a COVID-19 vaccination during the first month after the vaccine was recommended. In comparison, vaccination coverage among children aged 5–11 years was 20.7% during the first month after the recommendation (*6*). This report also identified early indications of racial and ethnic and urban versus rural differences in coverage previously observed for children in other age groups during the first months after the recommendations (*7*).

Approximately 5 months have elapsed since COVID-19 vaccination of children aged 6 months–4 years was recommended; this report indicates that a large proportion of children have parents who intend to have them vaccinated, yet these parents have concerns about vaccine safety. Confidence in vaccine safety varied by race or ethnicity and household income. Trusted persons (e.g., a child’s pediatrician) providing accurate information to parents about vaccine safety, especially to parents of Hispanic or Black children or parents in lower-income households, might encourage these parents to have their child vaccinated.

This report indicates that approximately three fourths of vaccinated children aged 6 months–4 years received their COVID-19 vaccine at a medical place. This is a larger proportion than that for children and adolescents aged 5–17 years, 38% of whom were vaccinated at medical places and 45% at pharmacies (*8*). The larger role of the medical home,^¶¶^ and medical places in general, in the delivery of vaccines to young children underscores the need for provider recommendation for vaccination. Studies have determined the importance of strong provider recommendations for vaccination (*9*), yet only approximately one fourth of unvaccinated children with parents open to vaccination were reported to have received a provider recommendation. A majority of parents open to vaccination consider the vaccine to be important; the addition of a provider recommendation that includes accurate information about vaccine safety could be critical to these parents deciding to have their children vaccinated.

The findings in this report are subject to at least two limitations. First, the response rate was 20%. Survey weights were calibrated to COVID-19 vaccine administration data to mitigate possible bias from incomplete sample frame, nonresponse, and misclassification of vaccination status; however, bias in estimates might remain after weighting. Second, vaccination status and intent to vaccinate were parent-reported and subject to recall and social desirability biases.

These findings indicate that a large proportion of unvaccinated children have parents who are open to vaccination; however, many parents had concerns about vaccine safety and had not received a provider recommendation. A strong vaccination recommendation from a trusted health care provider, along with accurate information about the safety of COVID-19 vaccination, could potentially increase COVID-19 vaccination coverage among young children.

**Summary**

**What is already known about this topic?**

Although COVID-19 vaccines are safe and effective, administrative data reported to CDC indicate that COVID-19 vaccination coverage among children aged <5 years is low.

**What is added by this report?**

Four percent of children aged 6 months–4 years had received ≥1 doses of COVID-19 vaccine based on interviews conducted during July 2022; 59% were unvaccinated, but the parent was open to vaccinating their child; and 37% were unvaccinated and the parent was reluctant to vaccinate. Among parents open to vaccination, 25% reported receiving a provider recommendation, and 57% were confident of the vaccine’s safety; confidence of vaccine safety varied by race or ethnicity and household income.

**What are the implications for public health?**

Health care provider recommendations and assurances of COVID-19 vaccine safety by trusted persons could increase vaccination coverage among young children.

## Footnotes

* Reported vaccination coverage estimates are by single year of age and, despite the vaccination recommendation targeting children aged 6 months to <5 years, population estimates for all children aged <5 years were used as the denominator. https://covid.cdc.gov/covid-data-tracker/#vaccination-demographic; https://covid.cdc.gov/covid-data-tracker/#vaccination-demographics-trends

† On July 22, 2021, NIS-CCM began including households with adolescents aged 13–17 years and asking the respondent about the adolescent’s COVID-19 vaccination status and intention to vaccinate the adolescent. On October 1, 2021, children aged 12 years and children aged 5–11 years (intent questions only) were added, and on November 2, 2021, vaccination status questions were added for children aged 5–11 years. In December 2021, the survey was expanded to include children aged 6 months–4 years (intention question only), and on June 19, 2022, vaccination status questions were added for children aged 6 months–4 years. https://www.cdc.gov/vaccines/imz-managers/nis/about.html#nis-ccm

§ The place-of-vaccination survey question was, “At what kind of place did [child] get [his/her] most recent COVID-19 vaccination?” Responses were coded by the interviewer into the following categories: doctor’s office, health department, clinic or health center, hospital, other medically related place, mass vaccination place, pharmacy or drugstore, workplace, elementary/middle/high school, or other nonmedically related place.

¶ The BeSD survey questions were 1) “How concerned are you about [child’s name] getting COVID-19?” (“Not at all concerned, a little concerned, moderately concerned, or very concerned.”); 2) “How important do you think getting a COVID-19 vaccine is to protect [child’s name] against COVID-19?” (“Not at all important, a little important, somewhat important, or very important.”); 3) “How safe do you think a COVID-19 vaccine is for [child’s name]?“ (“Not at all safe, somewhat safe, very safe, or completely safe.”); 4) “If you had to guess, about how many of your family and friends have gotten a COVID-19 vaccine for their children aged 6 months–4 years?” (“None, some, many, or almost all.”); 5) “Has a doctor, nurse, or another health professional ever recommended that you get a COVID-19 vaccine for [child’s name]” (“Yes or no.”); and 6) “How difficult would it be/was it for you to get [child’s name] a COVID-19 vaccine?” (“Not at all difficult, a little difficult, somewhat difficult, or very difficult.”).

** Parent interviews for 4,548 children aged 6 months–4 years were completed; among these interviews, 52 were excluded from the analyses (28 respondents didn’t know or refused to disclose vaccination status, and vaccination intent for 24 unvaccinated children was not reported) for a resulting analytic sample of 4,496. Vaccination status was assessed as of the date of the interview. Vaccination coverage estimates are based on interviews conducted during July 1–29, 2022, and therefore represent coverage as of approximately the midpoint of the survey fielding dates. Estimates for December 2021–June 2022 are from NIS-CCM interviews conducted during November 28, 2021–June 30, 2022.

†† The NIS-CCM survey weights adjusted for nonresponse and households without mobile telephones and were calibrated to match counts of children aged 6 months–4 years within each HHS region who had received ≥1 dose of COVID-19 vaccine as of midmonth, by sex, using data reported to CDC by jurisdictions.

§§ 45 C.F.R. part 46.102(l)(2); 21 C.F.R. part 56; 42 U.S.C. Sect. 241(d); 5 U.S.C. Sect. 552a; 44 U.S.C. Sect. 3501 et seq.

¶¶
https://www.aap.org/en/practice-management/medical-home/medical-home-overview/what-is-medical-home/

## References

[1] Hause AM, Marquez P, Zhang B, COVID-19 mRNA vaccine safety among children aged 6 months–5 years—United States, June 18, 2022–August 21, 2022. MMWR Morb Mortal Wkly Rep 2022;71:1115–20. 10.15585/mmwr.mm7135a3 36048728 PMC9472776

[2] Fleming-Dutra KE, Wallace M, Moulia DL, Interim recommendations of the Advisory Committee on Immunization Practices for use of Moderna and Pfizer-BioNTech COVID-19 vaccines in children aged 6 months–5 years—United States, June 2022. MMWR Morb Mortal Wkly Rep 2022;71:859–68. 10.15585/mmwr.mm7126e2 35771731

[3] CDC. About the National Immunization Surveys (NIS). Atlanta, GA: US Department of Health and Human Services, CDC; 2022. Accessed March 7, 2022. https://www.cdc.gov/vaccines/imz-managers/nis/about.html

[4] Wolter KK, Smith PJ, Khare M, Statistical methodology of the National Immunization Survey, 2005–2014. Vital Health Stat 1 2017;61:1–107. 29466229

[5] Brewer NT, Chapman GB, Rothman AJ, Leask J, Kempe A. Increasing vaccination: putting psychological science into action. Psychol Sci Public Interest 2017;18:149–207. 10.1177/1529100618760521 29611455

[6] Santibanez TA, Lendon JP, Singleton JA, Factors associated with receipt and parental intent for COVID-19 vaccination of children ages 5–11 years. medRxiv [Preprint posted online June 27, 2022]. https://www.medrxiv.org/content/10.1101/2022.06.24.22276865v1

[7] Murthy NC, Zell E, Fast HE, Disparities in first dose COVID-19 vaccination coverage among children 5–11 years of age, United States. Emerg Infect Dis 2022;28:986–9. 10.3201/eid2805.220166 35226801 PMC9045440

[8] Santibanez TA, Black CL, Vogt TM, Where are children ages 5–17 years receiving their COVID-19 vaccinations? Variations over time and by sociodemographic characteristics, United States. Vaccine 2022;40:6917–23. 10.1016/j.vaccine.2022.10.025 36280560 PMC9581793

[9] CDC. Influenza (flu): make a strong influenza vaccine recommendation. Atlanta, GA: US Department of Health and Human Services, CDC; 2022. Accessed September 8, 2022. https://www.cdc.gov/flu/professionals/vaccination/flu-vaccine-recommendation.htm

